# Supplementary material for: A study on phonemes recognition method for Mandarin pronunciation based on improved Zipformer-RNN-T(Pruned) modeling
Source: PLoS One. 2025 May 23;20(5):e0324048. doi: 10.1371/journal.pone.0324048 (PMC12101685; doi:10.1371/journal.pone.0324048)
Supplement: Attachment — Submitted filename: Response-to-Reviewers.pdf [file pone.0324048.s001.pdf]

Original Manuscript ID: PONE-D-24-60568

Original Article Title: "A Study on Phonemes Recognition Method for Mandarin Pronunciation Based on Improved Zipformer-RNN-T(Pruned) Modeling"

To: PLoS One Editor

Re: Response to reviewers

Dear Editor,

Thank you for allowing us to resubmit the manuscript and for the opportunity to address the reviewers' comments. We have carefully revised the manuscript based on the feedback provided in the attached summary of reviewers' comments (as shared by your office).

We are now uploading the following documents:

1. A point-by-point response to the reviewers' comments (under "Response to Reviewers"), addressing each concern raised in the attached summary.
2. A highlighted version of the revised manuscript (under "Highlighted PDF"), with all changes marked in yellow.
3. A clean version of the revised manuscript (under "Main Manuscript").

Please let us know if any further adjustments are needed. We appreciate your time and consideration.

Best regards,

Zhaohui Du et al

We sincerely appreciate the opportunity to revise our manuscript titled "A Study on Phonemes Recognition Method for Mandarin Pronunciation Based on Improved Zipformer-RNN-T(Pruned) Modeling" and are grateful for the meaningful comments provided by the reviewers. These comments have been invaluable in improving and refining our paper and have provided important guidance for our research. Below, we provide detailed responses to each of the reviewers' comments. Additionally, we have made comprehensive revisions to the manuscript. We have made every effort to clearly explain all the changes, and we hope the revised manuscript meets the publication requirements.

Reviewer#1, Concern # 1 (please list here): The introduction lacks clear logic and fails to effectively argue the need for the proposed model.

Author response: We thank the reviewers for pointing out the issue. We have structurally reorganised the Introduction to further explicitly present the causal relationship between the research motivation, technical challenges and model design of this paper's work. Specifically, we add the trade-off dilemma between the number of parameters, recognition accuracy, and real-time performance of traditional models at the end of the second paragraph of the Introduction section, and introduce our proposed lightweight and efficient model design on this basis to highlight the necessity and targeted improvement of the proposed model.

Author action:

Phoneme recognition is the process of identifying phoneme units using advanced speech recognition technology. In recent years, the significant advancements in speech recognition technology have been largely driven by the development of deep learning. With the introduction of deep neural networks (DNN) [4] and convolutional neural networks (CNN) [5], the ability of speech recognition systems to model speech time-series information has been significantly enhanced. In addition, the introduction of end-to-end models such as CTC [6], RNN-T [7], and LAS [8] has further simplified the complexity of the models, enabling speech recognition systems to exhibit stronger performance when processing long sequences. With the advancement of speech recognition technology, phoneme recognition technology has also been correspondingly improved. Researchers have suggested various deep learning-based phoneme recognition models, such as temporal modeling methods based on recurrent neural networks (RNN) [9], and self-attention mechanisms based on Transformers [10]. Despite the significant advancements made by the aforementioned methods in the field of speech recognition, their adaptability to Chinese phoneme recognition tasks remains inadequate. Existing mainstream models often have large parameter counts and complex structures, making them difficult to deploy in practical environments that require high resource efficiency and quick response times, such as language learning applications, online evaluation platforms, and mobile devices. Therefore, there is an urgent need to develop a lightweight model that combines strong modeling capabilities with a low real-time factor, which is crucial for the advancement of Chinese phoneme recognition systems.

Reviewer#1, Concern # 2 (please list here): The challenges of phoneme recognition are discussed, but it is not made clear why the Zipformer variant was chosen.

Author response: We thank the reviewers for their valuable comments. We agree that the description of the phoneme recognition challenge in the original manuscript, although detailed, was insufficiently elaborated in terms of the motivation for the model selection, and lacked an explanation of the connection between the Zipformer model and the specific task requirements. In the revised manuscript (fourth paragraph of the introduction in Chapter 1), we have added the following to further clarify the rationale for the selection and applicability of the Zipformer model.

Author action:

To address these challenges, this paper adopts the Zipformer architecture, which has shown outstanding performance in the field of speech recognition in recent years. Compared to the traditional Transformer, Zipformer significantly reduces computational complexity while maintaining its ability to model temporal information through a time-compression mechanism. In contrast to RNN-based models, it demonstrates higher efficiency in handling long-range dependencies and parallel computation. Additionally, Zipformer's multi-layer compression and decompression structure can accommodate the modeling needs of multi-dimensional information such as consonants, vowels, and tones in Chinese pronunciation, helping to capture subtle phonetic differences and trends. Moreover, Zipformer's modular structure and excellent scalability make it easier to implement fast inference in online pronunciation evaluation systems, meeting the requirements for real-time feedback. Therefore, in the task of Chinese phoneme recognition, Zipformer strikes a superior balance in terms of accuracy, latency, and model size compared to other mainstream models, making it the preferred model framework for this research.

Reviewer#1, Concern # 3 (please list here): The importance of RTF (Real Time Factor) optimisation in Chinese phoneme recognition is not adequately supported, lacking concrete examples or detailed reasoning.

Author response: We thank the reviewers for their questions. We have added in the introduction the strong reliance on real-time in Chinese phoneme recognition scenarios, especially in practical systems such as spoken language assessment and online education, where pronunciation analysis and feedback must be done in a very short time to ensure a natural and smooth system response.

Author action:

Currently, academic research primarily focuses on phoneme recognition for English and other Western languages, while research on Chinese phoneme recognition is relatively limited. This situation has resulted in a scarcity of high-quality datasets specifically for Chinese phoneme recognition. Compared to other languages, the phonetic system of Chinese is significantly more complex. Firstly, Chinese contains a large number of consonants and vowels that are phonetically similar, which makes them difficult to distinguish and prone to confusion. Secondly, tones play a crucial role in the semantic structure of Chinese, with the same syllable having completely different meanings depending on the tone [11]. For example, "ma" represents "mother" "hemp" "horse", and "scold" in the first to fourth tones, respectively. This reliance on tones to distinguish meanings greatly increases the difficulty of fine-grained modeling in Chinese phoneme recognition systems. Meanwhile, in practical application scenarios such as language learning and speech evaluation platforms, Chinese phoneme recognition systems also face the demand for strong real-time feedback. Learners typically expect immediate feedback on their pronunciation as soon as they finish speaking, so they can promptly adjust their pronunciation. This human-computer interaction scenario imposes a very low real-time factor (RTF) requirement on the system to ensure a smooth and natural user experience. Therefore, Chinese phoneme recognition tasks face

---

Reviewer#1, Concern # 4 (please list here): There is no clear correlation between tonal variations and the need for real-time processing in relation to the proposed method.

Author response: We fully understand the reviewers' concerns. Semantic differences in Chinese speech are often based on tones, which are acoustically represented as weak but complex fundamental frequency variations, posing a double challenge to system modelling capability and response speed. In this revision, we clarify the impact of tones on modelling accuracy and time delay, and further illustrate that Zipformer, with its hierarchical modelling capability and frame-level compression mechanism, is able to efficiently capture tonal variations while controlling inference delays, thus adapting to the specific challenges of Chinese speech. This has been added and highlighted in the introduction. This need drove the choice of Zipformer as a model architecture that excels in time efficiency. The relevant modifications have been clearly labelled in the third and fourth paragraphs of the introduction.

Author action:

Therefore,Chinesephonemerecognitiontasksfacetwo main challenges: first, the high requirement for modeling accuracy due to subtle phonetic differences such as tones; second, the strict limitations on inference speed and response delay in application scenarios. This means that phoneme recognition models must not only have the ability to model complex phonetic features accurately but also complete fast inference and output within a limited time budget to maintain efficient interactivity and practicality.

To address these challenges, this paper adopts the Zipformer architecture, which has shown outstanding performance in the field of speech recognition in recent years. Compared to the traditional Transformer, Zipformer significantly reduces computational complexity while maintaining its ability to model temporal information through a time-compression mechanism. In contrast to RNN-based models, it demonstrates higher efficiency in handling long-range dependencies and parallel computation. Additionally, Zipformer's multi-layer compression and decompression structure can accommodate the modeling needs of multi-dimensional information such as consonants, vowels, and tones in Chinese pronunciation, helping to capture subtle phonetic differences and trends. Moreover, Zipformer's modular structure and excellent scalability make it easier to implement fast inference in online pronunciation evaluation systems, meeting the requirements for real-time feedback. Therefore, in the task of Chinese phoneme recognition, Zipformer strikes a superior balance in terms of accuracy, latency, and model size compared to other mainstream models, making it the preferred model framework for this research.

Reviewer#1, Concern # 5 (please list here): Using only the AISHELL1-PHONEME dataset, the performance of the model in other linguistic environments (e.g. conversational Mandarin, dialects) was not validated, and model generalisation is questionable.

Author response: We thank the reviewers for their valuable comments. Regarding the issue of 'model generalisation has not been verified in more language environments', we not only use the AISHELL1-PHONEME dataset, but also introduce the ST-CMDS-PHONEME dataset for the comparative experiments in this paper, which is a set of speech data that covers more speech styles (e.g., spoken questions and answers, more emotion fluctuations) than AISHELL1. ST-CMDS is a set of speech data that covers a wider range of speech styles (e.g., spoken Q&A, more emotional fluctuations), and is closer to real application scenarios than AISHELL1. Our experimental results on both datasets consistently show that the proposed Zipformer-RNN-T(Pruned) model achieves the lowest recognition error rate and the fastest inference speed, showing good robustness and migration capability.

Author action:

The experiments in this study primarily use the Mandarin speech datasets AISHELL1 and ST-CMDS. The AISHELL1 dataset was originally recorded by 400 speakers from different regions of China using a variety of devices, including high-fidelity microphones, Android phones, and iOS phones, with an audio sampling rate of 16 kHz. The ST-CMDS dataset is an open-source Mandarin Chinese speech dataset primarily used for tasks such as speech recognition, speech synthesis, and speaker recognition. The dataset contains a large number of monophonic audio files with a 16 kHz sampling rate, covering various daily conversation scenarios, such as online chatting and voice command for smart devices.

Table 2. The division of the ST-CMDS-PHONEME dataset.

Data	Time(h)	Phoneme Quantity
Train	85	1900432
Dev	10	223750
Test	5	111618

As shown in Table 5, on the ST-CMDS-PHONEME dataset, Zipformer-RNN-T(Pruned) achieves a Dev WER of 4.28% and a Test WER of 4.51%, outperforming all other comparison models. Especially when compared to Transformer-CTC (Test WER 15.77%), Conformer-CTC (Test WER 6.47%), and Zipformer-CTC (Test WER 6.43%), Zipformer-RNN-T(Pruned) performs excellently in terms of accuracy. Although its Dev WER (4.81%) and Test WER (4.97%) are not significantly different from those of Conformer-RNN-T(Pruned), Zipformer-RNN-T(Pruned) still holds an advantage in inference speed (RTF of 0.002) and model size (61.1M parameters), demonstrating a balanced trade-off between efficiency and performance.

Table 5. Comparative Experimental Results of Mainstream Models on ST-CMDS-PHONEME Dataset.

Model	Dev	Test	RTF	Params
Conformer-CTC	6.22%	6.47%	0.029	108.7
Transformer-CTC	15.02%	15.77%	0.020	70.7
Zipformer-CTC	6.10%	6.43%	0.018	90.4
Zipformer-RNN-T	5.74%	5.94%	0.010	67.4
Conformer-RNN-T	4.98%	5.11%	0.011	83.7
Transformer-RNN-T(Pruned)	11.73%	12.38%	0.003	66.4
Conformer-RNN-T(Pruned)	4.81%	4.97%	0.003	78.1
Zipformer-RNN-T(Pruned)	**4.28%**	**4.51%**	**0.002**	**61.1**

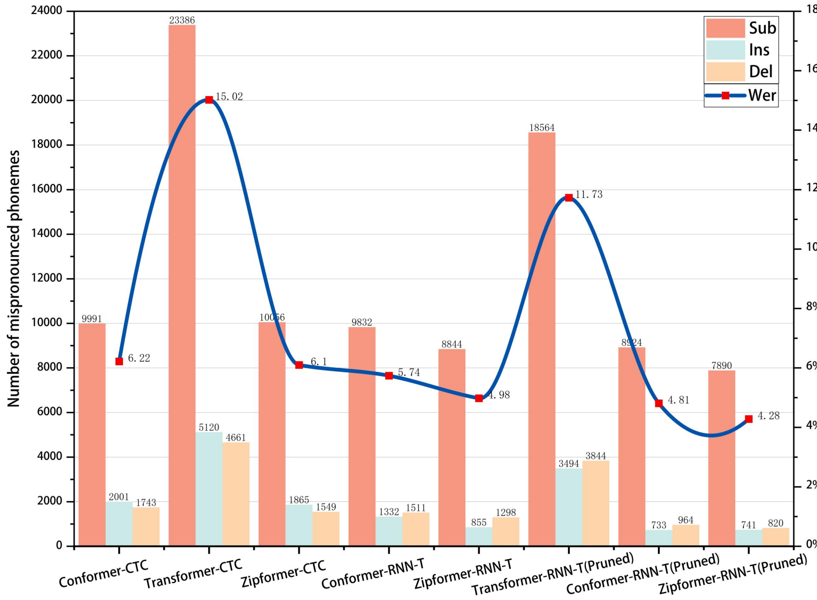

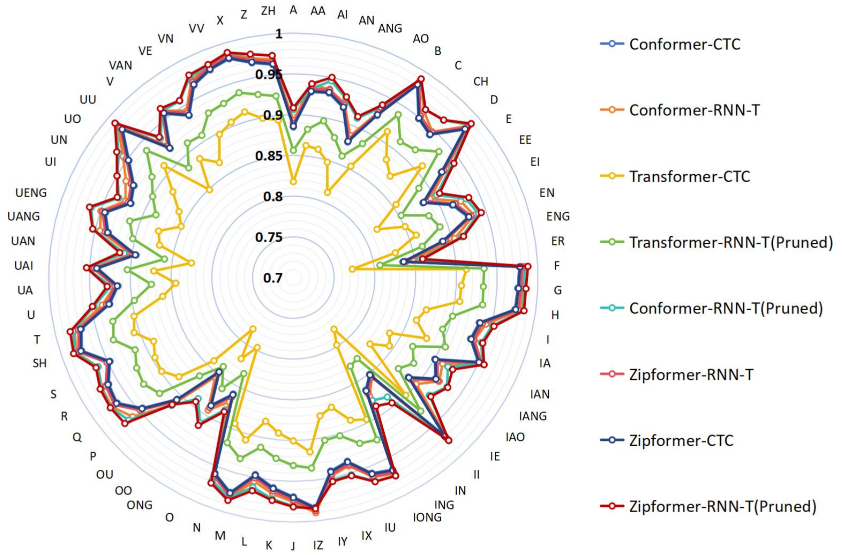

Table 7. Comparison of Model Performance Under Different Data Augmentation Strategies on the ST-CMDS-PHONEME Dataset.

Model	Train+			Train			Para
	Dev+	Test+	RTF	Dev+	Test+	RTF	
Conformer-CTC	6.38%	6.75%	0.025	6.84%	7.16%	0.028	108.7
Transformer-CTC	15.37%	15.99%	0.022	16.21%	16.93%	0.021	70.7
Zipformer-CTC	6.44%	6.78%	0.016	6.72%	6.97%	0.016	90.4
Zipformer-RNN-T	5.88%	6.15%	0.005	6.25%	6.48%	0.005	67.4
Conformer-RNN-T	5.37%	5.42%	0.004	5.55%	5.74%	0.008	83.7
Transformer-RNN-T(Pruned)	12.55%	13.18%	0.005	12.97%	13.49%	0.005	66.4
Conformer-RNN-T(Pruned)	4.98%	5.09%	0.004	5.34%	5.53%	0.004	78.1
Zipformer-RNN-T(Pruned)	**4.39%**	**4.54%**	**0.003**	**4.66%**	**4.90%**	**0.003**	**61.1**

We would like to once again express our sincere gratitude to all the reviewers for their valuable comments and suggestions. We have carefully considered each point and made thorough revisions to improve the quality and clarity of the manuscript. We believe that the manuscript has been significantly enhanced with your insightful feedback. We respectfully invite you to review the revised version and sincerely hope it meets the requirements for publication. Thank you very much for your time and consideration.
